# Observations on the Structure and Diseases of the Testis

**Published:** 1841-10

**Authors:** 


Art. V.—*Observations on the Structure and Diseases of the Testis.* By Sir Astley Cooper, Bart., f.r.s. Second Edition. Edited by Bransby B. Cooper, f.r.s. *With Twenty-four Coloured Plates.—London,* 1841. 4to, pp. 330.

The republication of this splendid volume supplies a want that has been long severely felt from the exhaustion of the first edition of it. With the exception of "some new matter upon the subject of varicocele," by the author, and the adoption of a "somewhat more perfect arrangement" of the old materials, and the addition of a new preface by the editor, the work is precisely such as it appeared originally from the hand of its lamented author; and assuredly, as the editor well observes, "it reflects no inconsiderable merit on Sir Astley Cooper's industry and acuteness, that although a period of ten years has elapsed since the first appearance of the work, a period, too, in which there has been no lack of labour in the field of anatomical and surgical science, no new facts of importance have been advanced on this subject." The extraordinary merits of this treatise have been so long and so universally acknowledged that it would be a work of supererogation to represent them in our pages: we will only say here, that the practical surgeon who is not master of its contents cannot be fully aware of the imperfection of his own knowledge on the subject of diseases of the testicle. No surgical library can be complete without it.

The preface to the volume does credit to the feelings of the editor; we trust that, ere long, we shall be indebted to him for a detailed account of the life and writings of his distinguished relative, in regard to whose character and conduct he but echoes the voice of the whole profession when he says: "In the exercise of his calling he exhibited an acute penetration, a solid judgment, a benevolent care, great suavity of address, and a most tender anxiety for the comfort, relief, and recovery of his patients to whatever rank of society they might belong." (Pref. viii.)

